# Advances in hiPSC‐Derived Brain Organoids as a Model to Study Neuroinflammation in Alzheimer's Disease

**DOI:** 10.1111/jnc.70416

**Published:** 2026-03-29

**Authors:** Esther Marques Alves Maciel, Nathália Costa Silva, Luiz Gustavo Pontes Santos, Fabiola M. Ribeiro

**Affiliations:** ^1^ Laboratory of Neurobiochemistry–Department of Biochemistry and Immunology, Institute of Biological Sciences (ICB) Universidade Federal de Minas Gerais Belo Horizonte MG Brazil

**Keywords:** Alzheimer's disease, astrocytes, cerebral organoid, human induced pluripotent stem cells, microglia, neuroinflammation

## Abstract

Neuroinflammation plays a fundamental role in several neurodegenerative diseases, including Alzheimer's disease (AD), the leading cause of dementia worldwide. As the main defense response of the central nervous system (CNS), neuroinflammation can be either protective or detrimental depending on the stage of the disease. The pivotal role of neuroinflammation in AD has led to increasing investigations into neuroinflammatory mechanisms, aiming to develop AD‐modifying therapies. A significant advance in the field was the emergence of the human induced pluripotent stem cell (hiPSC) model, enabling the study of patient‐derived cells. Moreover, the development of hiPSC‐derived brain organoids, which mimic specific aspects of the human CNS, has expanded our understanding of neuroinflammation in AD. Here, we review how AD organoid models have evolved, focusing on the integration of microglia—the brain's primary immune surveillance cells. We also summarize recent findings on how glial activation and the crosstalk between microglia and other CNS cells affect AD progression. Lastly, we address the potential of hiPSC‐derived organoids as a preclinical model for screening AD drugs.

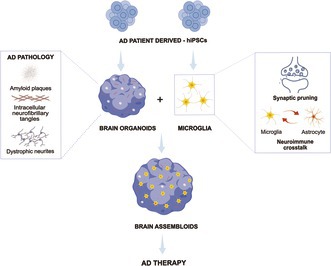

## Graphical abstract

Brain organoids and assembloids derived from human induced pluripotent stem cells (hiPSCs) represent a striking advance in biomedical research. These technologies allow a more accurate mimicry of the three‐dimensional cellular environment in vitro, enabling detailed investigations of Alzheimer's disease (AD) pathological features, such as β‐amyloid plaques, intracellular neurofibrillary tangles, and dystrophic neurites. They also provide a transformative platform for modeling key aspects of the disease, including neuroinflammation, and for supporting the development of new therapeutic strategies. Here we critically review the contribution of these 3D cell models to advance mechanistic insights and translational research in AD.

**Abbreviations**
AD: Alzheimer's disease
APOE: apolipoprotein E
APOECh: APOE Christchurch
APP: amyloid precursor protein
Aβ: amyloid‐beta
BBB: blood–brain barrier
CNS: central nervous system
DAM: disease‐associated microglia
fAD: familiar Alzheimer's disease
GSK3β: glycogen synthase kinase 3β
hESCs: human embryonic stem cells
hiPSC: human induced pluripotent stem cell
iMGLs: microglial‐like cells
NFTs: neurofibrillary tangles
NMDA: N‐methyl‐D‐aspartate
NRF2: nuclear factor erythroid 2‐related factor 2
PSCs: pluripotent stem cells
PSEN: presenilin
p‐tau: phosphorylated tau
sAD: sporadic Alzheimer's disease
TREM2: triggering receptor expressed on myeloid cells 2

## 1 Introduction

The study of neuroinflammation in Alzheimer's disease (AD) reached a milestone with the emergence of human induced pluripotent stem cells (hiPSCs), obtained by reprogramming differentiated human somatic cells. This technology opened up the possibility of directly exploring disease mechanisms in a patient‐specific manner, a significant advantage over animal models (Takahashi and Yamanaka 2006; Takahashi et al. 2007). Furthermore, the development of protocols for generating hiPSC‐derived brain organoids has allowed the recapitulation of pathological aspects related to the cortical environment, mimicking its regionalization and cellular diversity (Lancaster et al. 2013; Raja et al. 2016). Subsequently, the insertion of hiPSC‐derived microglia refined the model, expanding its applications (Abud et al. 2017; Ormel et al. 2018). It became feasible to investigate the association between human microglial function, genetic risk factors, and the burden of pathological molecules in AD organoids. Despite methodological advances, unresolved questions remain about how cellular interactions drive neuroinflammation. Likewise, improvements in model reproducibility and scalability are needed for more effective drug screening (Abud et al. 2017). This review discusses the importance of neuroinflammation in AD and how the field has progressed in understanding this topic, with the advent of hiPSC‐derived organoids as a more clinically relevant model to uncover the primary role of microglia in AD.

### 1.1 hiPSCs‐Derived Brain Organoids

**FIGURE 1:**
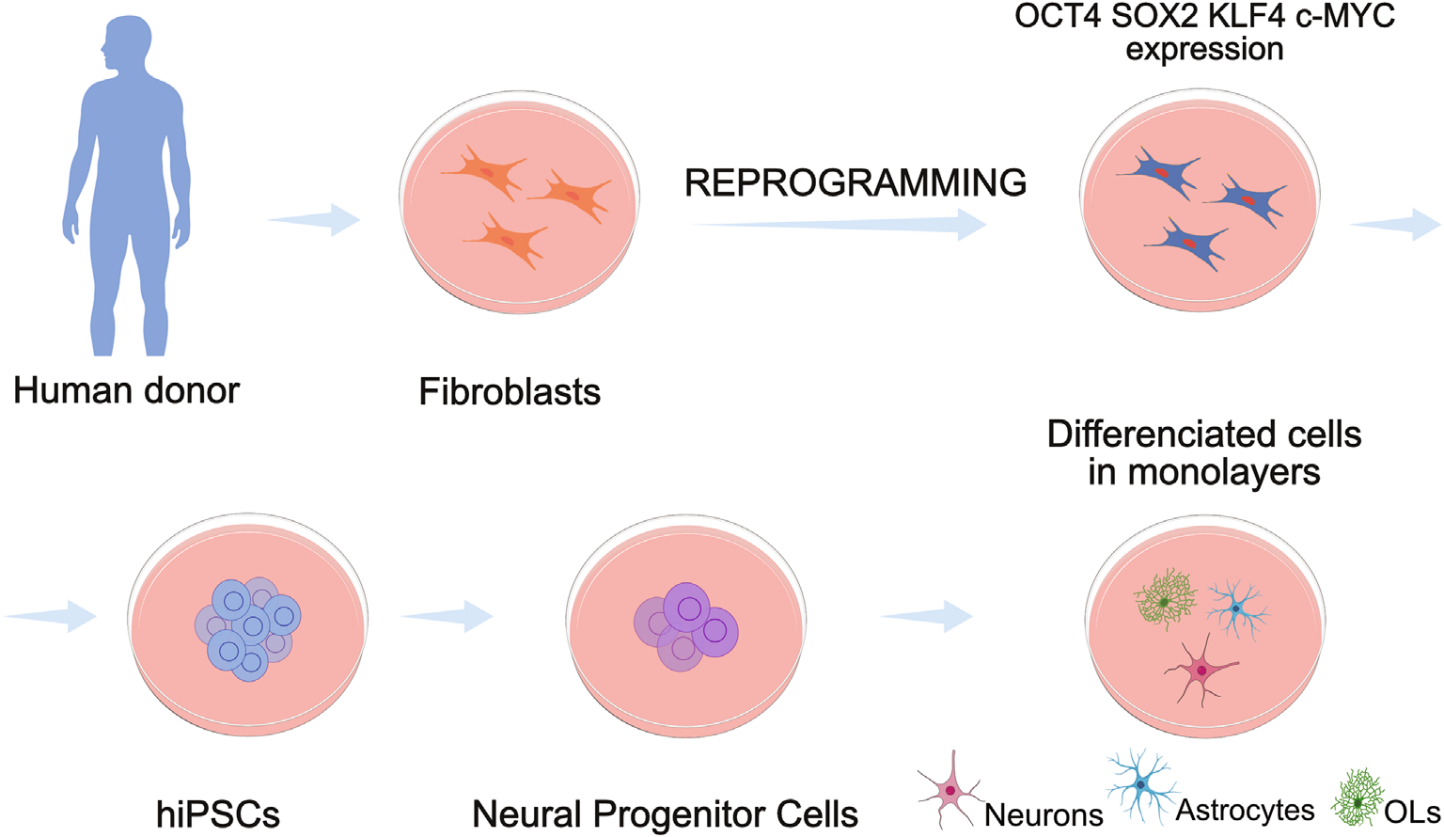
Generation of two‐dimensional neural cultures from human‐induced pluripotent stem cells (hiPSCs). Human somatic cells, such as fibroblasts, can be reprogrammed into hiPSCs through the ectopic expression of the Yamanaka factors (OCT4, SOX2, KLF4, and c‐MYC). Subsequently, neural progenitor cells can be differentiated into neurons, astrocytes, and oligodendrocytes (OLs) in monolayer culture.

Human pluripotent stem cells (PSCs) constitute self‐renewing populations capable of generating cells from all three embryonic germ layers. PSCs comprise human embryonic stem cells (hESCs) and hiPSCs (Zakrzewski et al. 2019). Obtaining hESCs is challenging and also involves ethical issues (Skottman et al. 2007). Thus, the advent of hiPSCs was a major breakthrough. It made possible the reprogramming of differentiated somatic cells into a pluripotent state through the ectopic expression of specific transcription factors—OCT4, SOX2, KLF4 and c‐Myc, as shown in Figure 1 (Takahashi and Yamanaka 2006; Takahashi et al. 2007). This innovation has advanced research in human neurodevelopment and disease modeling, as it allows the generation of patient‐derived cells modeling a wide range of pathological conditions (Wen et al. 2016; Casas et al. 2018; Kouroupi et al. 2020; Raja et al. 2022). Moreover, it addresses key limitations of animal models, such as interspecies differences in genetics, physiology and cellular responses, thus providing a more accurate and predictive platform for mechanistic studies, drug screening and regenerative medicine strategies (Lee et al. 2010; van der Worp et al. 2010; Martić‐Kehl et al. 2012; Sinnecker et al. 2014; Zakrzewski et al. 2019).

As cell development proceeds, the potential of PSCs becomes progressively restricted, giving rise to multipotent progenitors that generate the specialized cell types of specific organs (Hall and Watt 1989; Alison et al. 2002). Beyond the use in two‐dimensional systems, hiPSCs can also self‐organize into free‐floating aggregates in vitro, forming 3D cultures (Moscona and Moscona 1952; Watanabe et al. 2005; Takahashi and Yamanaka 2006; Yu et al. 2007; Eiraku et al. 2008). By controlling specific signaling pathways during induction, it is possible to recapitulate the cytoarchitecture of diverse tissues, including intestine, kidney, liver, and brain, among others (Sato et al. 2009; Spence et al. 2011; Lancaster et al. 2013; Takasato et al. 2016; Nguyen et al. 2021).

Cerebral organoids recapitulate early stages of human brain development and predominantly comprise neuroectoderm‐derived populations, including neural progenitor cells and neurons, astrocytes, and oligodendrocytes (Lancaster et al. 2013; Lancaster and Knoblich 2014; Paşca et al. 2015; Kim et al. 2019). Although microglia arise from mesoderm‐derived progenitors, these cells may spontaneously appear in organoids depending on the differentiation strategy employed. Moreover, microglial progenitors can either be co‐cultured with neural progenitor cells during organoid formation or incorporated after organoid maturation (for a detailed review, see Zhang et al. 2023) (Ormel et al. 2018; Popova et al. 2021; Zhang et al. 2023; Wenzel et al. 2024; Mrza et al. 2024). In this context, the integration of hiPSC‐derived microglial‐like cells further refined these systems, enabling the investigation of human microglial function, genetic risk factors, and the role of pathological proteins in AD organoid models (Park et al. 2018; Fagerlund et al. 2021; Sabate‐Soler et al. 2022).

hiPSCs can be grown freely, generating organoids that exhibit cells with different regional identities, such as forebrain, midbrain, hindbrain, retinal, and mesodermal lineages. Otherwise, when there is a specific interest in a particular brain region, protocols with more complex developmental cues can be used to reproduce early regionalization processes, modeling, for instance, key aspects of human cortical, hippocampal, and midbrain organization, among other brain regions (Lancaster et al. 2013; Lancaster and Knoblich 2014; Sakaguchi et al. 2015; Jo et al. 2016; Raja et al. 2016). It is likewise feasible to generate choroid plexus–like structures and organoids capable of secreting cerebrospinal fluid (Pellegrini, Bonfio, et al. 2020; Pellegrini, Albecka, et al. 2020).

More recently, researchers have fused organoids derived from distinct brain regions, giving rise to assembloids. These structures enable the reconstruction of long‐range neuronal circuits as well as interactions between neural and non‐neural tissues, such as muscle cells (Andersen et al. 2020; Miura et al. 2020). Another major advancement has been the development of vascularized brain organoids, which represents a critical step towards overcoming limitations related to nutrient diffusion, structural growth and the faithful recapitulation of brain architecture. Following these innovations, organoid models have begun to incorporate functional components of the blood‐ brain barrier (BBB) (Cakir et al. 2019; Dao et al. 2024). Together, such aspects represent a crucial step towards using in vitro models to investigate neuroinflammatory and neurodegenerative processes. Thus, here, we will also discuss the main findings in AD research using brain organoid and assembloid systems.

### 1.2 Alzheimer's Disease

AD affects more than 32 million people worldwide, accounting for about 70% of dementia cases. The affected population is even higher in earlier stages of the disease, with an estimated 69 million individuals living with prodromal AD and 315 million in the preclinical phase (Gustavsson et al. 2023). According to the Alzheimer (2024), AD is a debilitating and fatal disease, with increasing global prevalence and socioeconomic impact, for which there is currently no curative treatment: https://www.zotero.org/google‐docs/?enNTWB (Alzheimer 2024). Typically, AD therapy aims to manage neuropsychiatric symptoms and alleviate cognitive impairment. In recent years, the first disease‐modifying treatment was approved, employing passive immunization with anti‐amyloid β monoclonal antibodies. Although it is a promising approach, trials have demonstrated a limited effect on delaying cognitive and functional decline, raising questions about its clinical significance. Overall, while current treatments improve patients' quality of life to some extent, they have low effectiveness in modifying the AD course (Fox et al. 2025). Furthermore, immunotherapy can lead to severe adverse effects, which include cerebral edema and hemorrhage. These adverse events require clinical monitoring and may necessitate treatment adjustment or discontinuation (Hampel et al. 2023).

Thus far, AD etiology remains poorly understood. In < 5% of the cases, AD is caused by autosomal‐dominant mutations in the amyloid precursor protein (APP) or presenilin (PSEN) genes (Zhu et al. 2015). These mutations lead to an increased production of amyloid‐β peptide (Aβ) and an earlier manifestation of the disease, known as familial AD (fAD) or early‐onset AD. In patients with fAD, the mean age at onset is 47.3 years (Liu et al. 2025). However, the majority of the patients develop late‐onset AD, also known as sporadic AD (sAD), which is characterized by a multifactorial and not fully understood etiology (reviewed by Krishnamurthy et al. 2025). The main consensus is that sAD arises from a combination of several risk factors, classified as modifiable and non‐modifiable. Modifiable factors include environmental and lifestyle aspects, such as physical activity, social and cognitive engagement, smoking, and chronic conditions, including hypertension, diabetes, and cardiovascular disease. Among non‐modifiable factors, aging is the strongest risk factor for sAD, which typically manifests after the age of 65. Female sex, family history, ε4 allele of apolipoprotein E (APOE4), and other polygenic risk factors also contribute to disease development (Livingston et al. 2024; Krishnamurthy et al. 2025).

AD is characterized by progressive cognitive decline linked to specific neuropathological changes that accumulate over time (Jack et al. 2024). These abnormalities were first described by Alois Alzheimer as a peculiar illness of the cerebral cortex, associated with severe memory impairment, temporal and spatial disorientation, delirium, language deficits, as well as changes in thinking and behavior (Alzheimer 1907). His observations of brain atrophy and neuronal loss were later confirmed in other postmortem samples (Hyman et al. 1984; Terry et al. 1991). Additionally, the pathological deposits reported by Alzheimer were identified as extracellular plaques composed of Aβ and intracellular neurofibrillary tangles (NFTs) formed by the microtubule‐associated protein tau–both considered hallmarks of the disease (Glenner and Wong 1984; Grundke‐Iqbal et al. 1986).

There is a positive correlation between cognitive decline and loss of synaptic density (Dekosky and Scheff 1990; Terry et al. 1991). This synaptic loss develops alongside chronic synaptic dysfunction, which may be triggered by toxic Aβ and tau species, mainly in their soluble forms. Several mechanisms have been described linking toxic Aβ and tau species to neurodegeneration, including neuronal hyperactivity, deficits in axonal transport, and metabolic impairment (Tracy et al. 2022; Tzioras et al. 2023). In addition, neuroinflammation has been proposed as a key factor directly implicated in AD‐associated neurodegeneration and synaptic loss (Wilson et al. 2023).

### 1.3 Neuroinflammation in Alzheimer's Disease

**FIGURE 2:**
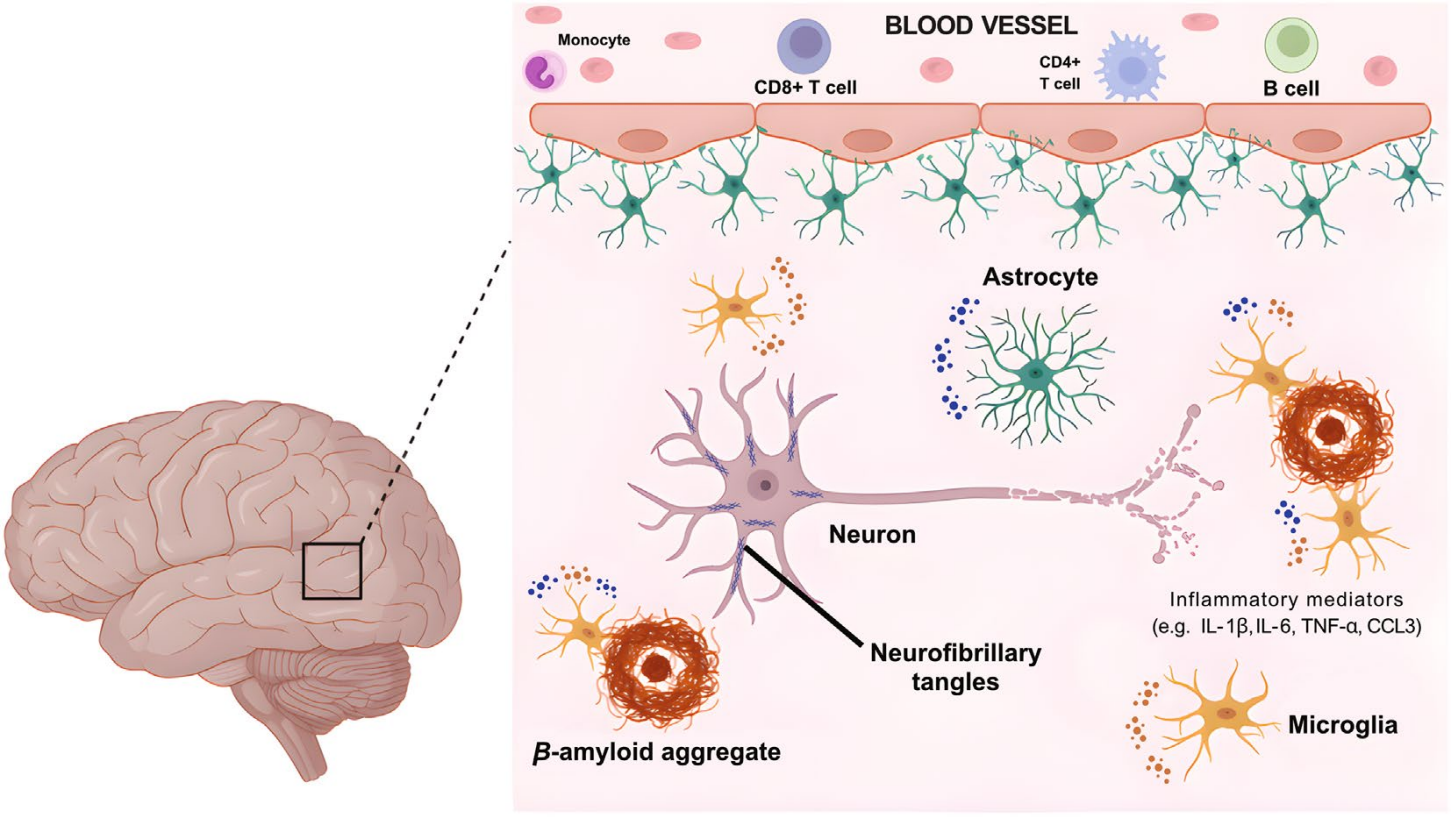
Neuroinflammatory activation in Alzheimer's disease (AD). Protein aggregates, such as amyloid‐β plaques and tau‐containing neurofibrillary tangles are major hallmarks of AD pathology. These protein aggregates activate resident immune cells of the central nervous system, such as microglia and astrocytes, that contribute to AD progression through the release of inflammatory mediators, such as cytokines (e.g., IL‐1β, IL‐6, TNF‐α) and chemokines (e.g., CCL‐3). As the disease progresses, neuronal damage occurs and, consequently, neurodegeneration. Alterations in the brain microvascular environment also arise, including increased blood–brain barrier permeability and infiltration of peripheral immune cells, such as monocytes and lymphocytes.

Neuroinflammation is a defense response mechanism of the central nervous system (CNS) to insults of external or internal origin, such as pathogens, trauma, or protein aggregates (Wyss‐Coray 2002). This process involves different cell types, including glia and endothelium cells, as well as peripheral immune cells that infiltrate the CNS, such as monocytes and lymphocytes. The production of inflammatory mediators give rise to distinct cellular activation states and may result in either a loss or gain of function (Lyman et al. 2014). Indeed, chronic glial reactivity is a factor that contributes to synaptic dysfunction and neurodegeneration in AD, with Aβ presence and tau phosphorylation exacerbating this process (Rohden et al. 2025), as represented in Figure 2.

In that sense, neuroinflammation can be detrimental, resulting in tissue damage, loss of homeostasis, and impaired functionality. However, it can also be resolutive, leading to the elimination of the offending agent, tissue repair, and neuroprotection. This balance primarily depends on the intensity and duration of neuroinflammatory processes, emerging from intricate signaling among different cell populations (Disabato et al. 2016). For instance, it can be mediated by Toll‐like receptors, receptors for advanced glycation end products, and neurotransmitter binding receptors, resulting in increased phagocytic functions and release of cytokines and chemokines by glial cells (Deczkowska et al. 2018; Li, Shui, et al. 2021).

Neuroinflammation is present in several neurodegenerative diseases and has been pointed out as a key factor directly involved in their progression (Wilson et al. 2023). Thus, understanding neuroinflammatory mechanisms can be useful for the development of disease‐modifying therapies, particularly for conditions with complex etiologies, such as AD. In this context, modulating the glial phenotype may have a neuroprotective effect by regulating aspects of CNS homeostasis, such as molecule clearance, BBB integrity, synapse pruning, and neurotransmitter balance (Kwon and Koh 2020).

From the early stages of AD, aberrant accumulation of Aβ and NFTs leads to the microglial transition from a homeostatic state to an inflammatory profile. This functional switch results in a unique phenotype termed disease‐associated microglia (DAM) that precedes significant cognitive impairment and correlates with the disease hallmarks (Edison et al. 2008; Keren‐Shaul et al. 2017; Dani et al. 2018). Microglia may initially play a protective role through the phagocytosis of cellular debris and amyloid aggregates. However, as the disease progresses, these cells adopt a neurotoxic state (El Khoury et al. 2007). It has been demonstrated that the triggering receptor expressed in myeloid cells (TREM2) appears to be involved in the beneficial effects of microglia, promoting recognition of damage‐associated molecules, microglial survival, and migration towards amyloid plaques, thereby favoring Aβ clearance (Wang et al. 2015; Lee et al. 2018). Accordingly, some TREM2 variants increase AD risk precisely by affecting microglial functions. For example, a study conducted on hiPSC‐derived microglia demonstrated that the R47H variant exacerbates synaptic phagocytosis, culminating in neuronal loss (Popescu et al. 2023). Overall, the course of glial phenotypic changes depends on genetic and environmental susceptibilities, which shape disease severity among individuals (Leng and Edison 2021).

## 2 hiPSCs As a Tool for Investigating Neuroinflammatory Mechanisms in AD

The hiPSC‐derived cells have been a useful tool to shed light on neuroinflammation and the critical role of microglia. The key reason is that, although human and murine microglia share core similarities, the genetic signature of human microglia differs from that of murine microglia, exhibiting higher expression of specific immune genes, such as complement factors and Toll‐like receptors (Galatro et al. 2017; Gosselin et al. 2017). Gene expression associated with cell proliferation also differs (Galatro et al. 2017). Moreover, microglial phenotypes diverge during inflammation in terms of immune signaling dynamics and metabolic reprogramming (Sabogal‐Guáqueta et al. 2023). In aging and AD, human microglia undergo changes that are not recapitulated by murine microglia, including transcriptional alterations linked to the chemoattraction of peripheral cells and APOE upregulation, as well as reduced microglial motility (Galatro et al. 2017; Friedman et al. 2018; Srinivasan et al. 2020).

Considering that hiPSCs are used to generate advanced models to study human cellular phenotypes, we will discuss the importance of their derived systems for exploring neuroinflammatory mechanisms in AD.

### 2.1 Dissecting Neuroimmune Crosstalk in AD Organoids

The traditional view of neuroinflammation in AD, centered on isolated microglial activation, has been replaced by a more complex model that includes other resident cells of the CNS and positions microglia–astrocyte signaling as a key mechanism of neurodegeneration. For instance, this bidirectional interaction appears to dictate the balance between neuroprotection and neurotoxicity, depending on the glial phenotypes adopted across the different stages of the disease (Chen, Xu, et al. 2025). Astrocytes play a crucial role in regulating BBB integrity, metabolic support, and synaptic plasticity. In response to insults, astrocytes may have neuroprotective effects, for example, by increasing the synthesis of neurotrophic factors (Linnerbauer and Rothhammer 2020; https://www.zotero.org/google‐docs/?DMztvY). However, under chronic inflammatory conditions, these astrocytic functions may be impaired, further exacerbating neurodegeneration (Lee et al. 2025). As with microglia, interspecies differences are an important consideration when examining the role of astrocytes in neuroinflammation. Human astrocytes exhibit more complex morphology and greater diversity of cortical subpopulations than murine astrocytes (Oberheim et al. 2006, 2009). Moreover, there are differences in the metabolic profile and astrocytic responses to neurotransmitters, inflammatory cytokines, and oxidative stress (Zhang et al. 2016; Li, Pan, et al. 2021). Likewise, human glial cells may respond differently to pharmacological compounds. Taken together, these particularities underscore the value of human models to study microglia–astrocyte crosstalk and to develop novel therapies targeting neuroinflammation.

In the context of AD, astrocyte priming boosts microglial Aβ clearance, a mechanism influenced by the APOE genotype and validated in cerebral organoids (Lee et al. 2025). Interestingly, astrocytes can regulate DAM gene signature, upregulating microglial expression of proteins such as TREM2 and APOE, as shown in hiPSC‐derived cultures (Lish et al. 2025). Astrocytes can also have a detrimental effect on microglial function. It has been shown in AD organoids that astrocytic production of interferon‐gamma (IFNγ) may inhibit antioxidant pathways in microglia, increasing their sensitivity to oxidative stress and promoting a pro‐inflammatory state (Kang et al. 2024). Glial dysregulation in AD also involves oligodendrocytes, the myelin‐producing cells of the CNS. Their dysfunction is associated with the emergence of disease hallmarks and has been proposed as an upstream factor in Aβ deposition (Braak and Braak 1996; Bartzokis 2011). Accordingly, oligodendrocytes undergo significant transcriptional changes in the human brain at early stages of AD, leading to amyloid production and impaired myelination (Gazestani et al. 2023; Badina et al. 2025). Although different protocols allow the efficient generation of myelinating oligodendrocytes in vitro, few studies have investigated their role in AD using three‐dimensional human models (Abud et al. 2017; Madhavan et al. 2018; Nzou et al. 2020; Ng et al. 2021). Recently, Ramirez et al. (2025) compared oligodendrocyte gene expression across APOE genotypes in a simplified three‐dimensional hiPSC‐derived spheroid model. They detected differences in myelin‐related transcripts, suggesting an impact on myelination capacity (Ramirez et al. 2025). Similar approaches hold promise for clarifying the interplay between oligodendrocyte homeostasis and AD progression (Cerneckis and Shi 2023).

In addition, peripheral immune cells participate in glial activation in AD. Using a cortical organoid microphysiological system, Tian et al. (2025) demonstrated the pathogenic role of human AD monocytes, which exhibited intense inflammatory responses and increased infiltration capacity. Through positive activation feedback, astrocytes and AD monocytes produced IL‐1β and CCL3, which, in turn, triggered neuronal apoptosis in organoids (Tian et al. 2025). Mechanisms of mutual activation between T lymphocytes and glia in AD have also been explored in microfluidic devices that combine 3D cultures with infiltrating peripheral cells (Jorfi et al. 2023). Despite being innovative, this system—like most in vitro approaches—is limited in recapitulating neuroimmune signaling, as this process also involves microglia and other cells from the brain microvascular environment. To address these limitations, Nzou et al. (2020) developed a more complex system recreating the human neurovascular unit for studying BBB dysfunction. In an inflammatory context, their multicellular model showed increased permeability, along with oxidative stress and the upregulation of cytokine and chemokine levels (Nzou et al. 2020). Crucially, in vitro and in vivo models should be able to faithfully recapitulate the neuropathological landscape observed in the human AD brain, reproducing the three core determinants: Aβ accumulation, hyperphosphorylated tau aggregation, and sustained neuroinflammation (Park et al. 2018; https://www.zotero.org/google‐docs/?d6SouG).

Consistent with the increasing complexity of three‐dimensional models, it has been reported that hiPSC‐derived AD organoids exhibit features typical of AD neuroinflammation, including inflammasome formation, oxidative stress, microglial senescence, and excessive synaptic pruning (Jin et al. 2022; Fertan et al. 2024). Further results also indicate that innate immune signaling pathways become activated in AD brain organoids, triggering apoptotic pathways and impairing neurogenesis (Scopa et al. 2023). Conversely, beneficial glial effects have been observed in this model. Takata et al. (2023) demonstrated that microglia can transition to a DAM‐like phenotype in Aβ‐treated organoids, undergoing transcriptional and morphological changes. Moreover, these cells can migrate and attenuate amyloid deposition, increasing neuronal viability (Takata et al. 2023). Additionally, in long‐term organoids, astrocytic support of microglia promoted their survival and function, which in turn culminated in enhanced synaptic density and reduced tau phosphorylation (Chen, Sun, et al. 2025). Altogether, these data indicate that brain organoids are suitable for evaluating diverse glial phenotypes in AD, as well as other neuroinflammatory parameters.

Another essential aspect underlying neuroinflammation explored in organoid and assembloid systems is the APOE genotype, which represents the major genetic risk factor for sAD. Studies have shown that the APOE genotype influences glial activation in humans, affecting cellular responses to inflammatory stimuli and protein aggregates in AD (Friedberg et al. 2020; Serrano‐Pozo et al. 2021). In line with this, Li, Martens, et al. (2025) demonstrated that transcriptomic changes in AD patients, associated with inflammatory signaling, synaptic function, and myelination, are APOE genotype–dependent. Although murine models remain widely used to investigate APOE effects in oligodendrocyte function and microglial reactivity, significant differences in APOE expression between humans and mice pose challenges for the understanding of AD pathophysiological mechanisms https://www.zotero.org/google‐docs/?PcD7So (Zhu et al. 2012; Blanchard et al. 2022; Holtzman et al. 1999; Fagan et al. 2002; Maloney et al. 2007; Hudry et al. 2013; Balu et al. 2019). Thus, to overcome interspecies differences, there is increasing interest in investigating APOE genotype effects in human models, especially in AD brain organoids. The main findings on this topic are discussed in the next section.

### 2.2 Revealing the Impact of APOE in AD Organoids

APOE is an important lipid carrier in the brain and is found in both NFTs and amyloid deposits (Namba et al. 1991). The APOE genotype is defined by the ε2, ε3, and ε4 alleles, which encode the APOE2, APOE3, and APOE4 protein isoforms in humans (Zannis and Breslow 1981; reviewed by Frisoni et al. 2022). APOE3 is the most prevalent isoform and is considered neutral regarding AD. APOE2 exerts a protective effect, whereas APOE4 considerably increases the risk of developing the disease, reduces the age of onset and intensifies the pathology severity (Corder et al. 1993; Strittmatter et al. 1993; Saunders et al. 1993; Corder et al. 1994). Under physiological conditions, astrocytes are the main source of cerebral APOE (Boyles et al. 1985). However, stress conditions can induce APOE synthesis by neurons and microglia, while reducing its expression by reactive astrocytes (Uchihara et al. 1995; Xu et al. 2006). For instance, APOE upregulation in microglia has been associated with Aβ and tau accumulation through transcriptomic analyses of human AD brains (Mathys et al. 2019). Thus, the mechanisms of APOE on AD pathology likely involve changes in the glial phenotype. Given that APOE effects are species‐ and isoform‐specific, hiPSC‐derived organoids have been useful for investigating this topic. This is particularly evident when these 3D cell models are combined with gene editing approaches to assess the impact of different human variants (Holtzman et al. 2000).

Lin et al. (2018) demonstrated that homozygous APOE4 causes transcriptional alterations in neurons and glial cells compared to APOE3. As a result, neurons exhibit synaptic hyperactivation, while astrocytes and microglia show impaired phagocytosis. In APP‐duplicated organoids with elevated Aβ production, the APOE4 genotype intensifies the accumulation of Aβ and phosphorylated tau (p‐tau), possibly by reduced glial clearance (Lin et al. 2018). Another study conducted by Park et al. (2024) confirmed that the APOE4 genotype impairs microglial phagocytosis by reducing the levels of TREM2. Consequently, microglia lose the ability to recognize externalized phosphatidylserine in dystrophic neurons in the vicinity of amyloid plaques, leading to reduced Aβ uptake in both hiPSC‐derived 2D co‐cultures and brain organoids (Park et al. 2024). These results are consistent with evidence showing that APOE4 impairs various neuronal and glial functions, ultimately leading to neurodegeneration (Blumenfeld et al. 2024).

To uncover the role of APOE4 in different cell types in a three‐dimensional environment, Huang et al. (2022) developed chimeric organoids with selective expression of APOE4 in neurons and/or astrocytes. They observed that the presence of APOE4 in either cell type was sufficient to induce lipid accumulation in neurons, showing a synergistic effect when co‐expressed. Conversely, APOE4 expression was necessary in both cell types for a detectable increase in neuronal p‐tau. Treatment with cholesterol synthesis inhibitors partially reversed this accumulation, indicating cholesterol metabolism as a target for AD‐associated tau pathology (Huang et al. 2022). Future studies may help to elucidate how cell‐type‐specific APOE mechanisms contribute to AD pathology across different disease stages.

Neuronal lipid burden has been identified as a convergent factor between APOE genotype and neurodegeneration. It has been demonstrated that APOE4 astrocytes induce neuronal hyperexcitability, leading to an increase in reactive oxygen species, including peroxidated lipids, and potentially triggering ferroptosis (Li, Benitez, et al. 2025). Another mechanism by which APOE can affect neuronal survival is through the regulation of autophagy. Using homozygous isogenic hiPSCs for APOE3 and APOE4, Park et al. (2022) showed that APOE4 reduces the levels of autophagy‐related molecules in both neurons and microglia within brain assembloids. Although the authors did not investigate how APOE4 causes this reduction, they highlight that this process may influence AD progression (Park et al. 2022).

hiPSC‐derived models have also been useful for studying protective APOE variants, such as APOE2 and the rare Christchurch variant (APOECh; R136S) (Wardell et al. 1987; Brookhouser et al. 2021; Ding et al. 2024). APOECh is associated with delayed clinical onset and reduced tau pathology in familial AD, despite substantial amyloid accumulation (Arboleda‐Velasquez et al. 2019). Interestingly, co‐culture with APOECh microglia has been shown to decrease p‐tau levels in PSEN1 mutant organoids. Researchers have reported that APOECh microglia are less susceptible to Aβ effects, being able to preserve the phagocytic activity necessary for tau clearance. They also observed resistance to Aβ‐driven lipid peroxidation, ferroptosis, and cytokine production in APOECh microglia (Sun et al. 2024).

**FIGURE 3:**
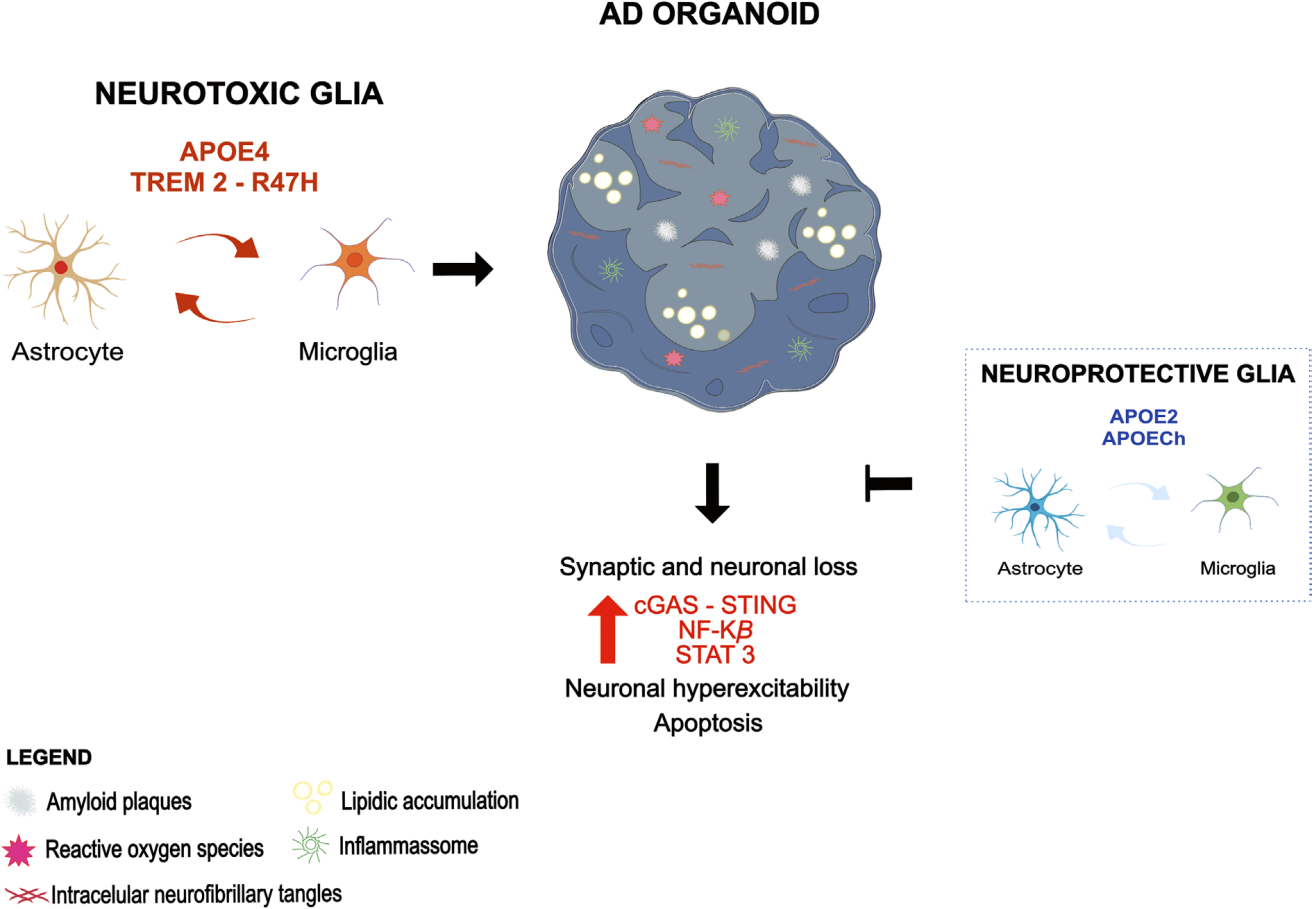
Apolipoprotein E (APOE) isoforms modify Alzheimer's disease (AD) pathology in organoids through glial modulation. APOE4 negatively affects the phenotype of astrocytes and microglia by altering lipid metabolism, activating inflammatory pathways, and reducing their phagocytic capacity. As a result, several pathological alterations are intensified in AD organoids, including oxidative stress, neuronal ferroptosis, synaptic dysfunction, as well as Aβ and tau accumulation. Protective variants of APOE, such as APOE2 and APOECh, act in the opposite manner.

It is thought that the Christchurch mutation reduces APOE affinity for cellular receptors such as heparan sulfate proteoglycans (Arboleda‐Velasquez et al. 2019). Indeed, treatment of APOE3 microglia with an APOE‐mimetic peptide to inhibit these molecular interactions recapitulates the APOECh effects (Sun et al. 2024). Another possible mechanism of APOECh was identified in AD organoids, where its expression was detected mainly in glial cells. The study observed that the variant enhances β‐catenin/Wnt signaling, which in turn inhibits tau phosphorylation (Perez‐Corredor et al. 2024). These results are consistent with data from murine AD models in which APOECh‐mediated attenuation of pathological processes was linked to microglial modulation (Chen et al. 2024; Tran et al. 2025). Taken together, the evidence supports the use of hiPSC‐derived brain organoids to investigate the neurotoxic and protective pathways linked to APOE variants and to identify therapeutic targets for AD. Figure 3 summarizes the main findings regarding APOE effects on glial phenotypes in AD organoids.

## 3 Expanding the Understanding of AD With Assembloids

**FIGURE 4:**
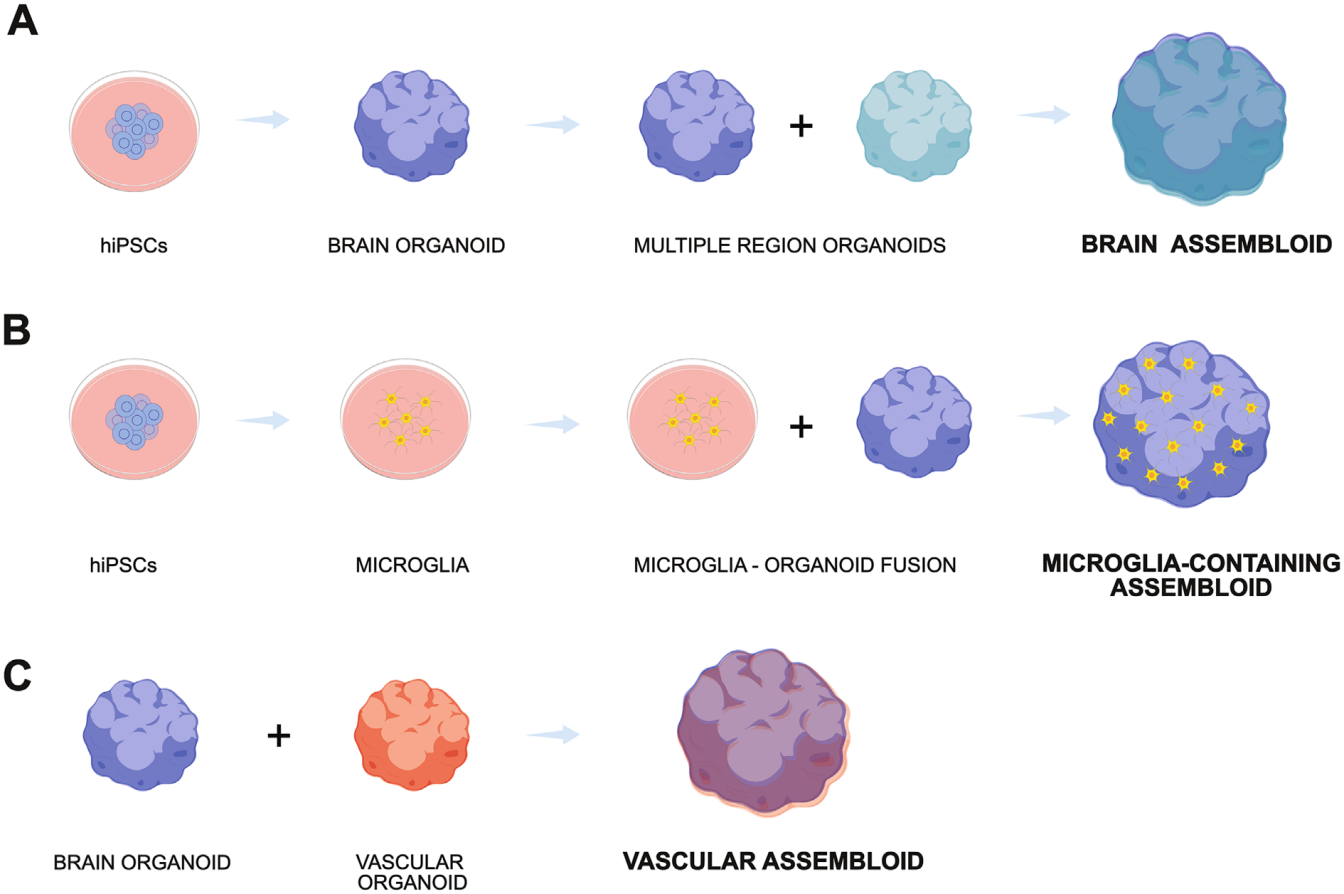
Three‐dimensional systems generated from human‐induced pluripotent stem cells (hiPSCs). (A) Three‐dimensional cultures derived from hiPSCs, such as organoids, exhibit enhanced cell–cell interaction and increased tissue complexity. Multiple brain‐region organoids can be combined, giving rise to assembloid models. (B) Incorporating microglia from Alzheimer's disease patients into hiPSC‐derived organoids generates microglia‐containing assembloids, enabling the study of neuroimmune interactions. (C) Fusing brain organoids with vascular organoids provides structural support and a more representative modeling of blood–brain barrier interactions, generating vascular assembloids.

Assembloids represent a major advance on three‐dimensional models as they enable the combination of distinct cell types and brain areas, giving rise to environments in which neurons, astrocytes, microglia and other cells establish spatially organized and functionally integrated interactions that more closely resemble human brain physiology, as illustrated in Figure 4; https://www.zotero.org/google‐docs/?lMjzPg (Andersen et al. 2020; Miura et al. 2020).

Assembloids can be generated by the junction of brain organoids and induced microglia‐like cells (iMGs) derived from hiPSCs from AD patients. Becerra‐Calixto et al. (2025) combined cerebral organoids with microglia from a familial AD patient carrying a PSEN2 familial mutation, creating a valuable disease‐modeling tool. These assembloids successfully recapitulate key AD features, including amyloid plaque‐like deposits and neurofibrillary tangle‐like structures. In this system, iMGs adopted a DAM phenotype, exhibiting reduced phagocytic capability and alterations in gene expression, consistent with chronic neuroinflammation (Becerra‐Calixto et al. 2025).

The degree of tissue architecture attained by assembloids can also allow the detection of cell–cell communication pathways that would remain undetectable in bidimensional cultures. Glycogen synthase kinase 3 beta (GSK3β) is a multifunctional kinase that, in the context of AD, contributes to abnormal tau phosphorylation, synaptic dysfunction and neuronal death (Hanger et al. 1992; Mandelkow et al. 1992; Avila et al. 2010). GSK3β also modulates NRF2, a transcription factor that drives antioxidant and cytoprotective gene expression (Rojo et al. 2008; Chauhan et al. 2022). Li, Benitez, et al. 2025 screened human assembloids composed of neurons, astrocytes and microglia using CRISPR interference to repress genes. Through this system, they demonstrated that GSK3β suppresses the NRF2‐mediated protective response to oxidative stress in neurons under high activity. This effect was observed within the three‐dimensional environment, being absent in two‐dimensional monocultures. These findings demonstrate that a more complex tissue‐like architecture involving neuron and glia interaction is required for the activation of GSK3β‐dependent control of the NRF2 pathway (Li, Benitez, et al. 2025).

Another frontier in assembloid technology is the generation of vascularized systems, which enable the investigation of interactions among neural and vascular cells (Naderi‐Meshkin et al. 2023). For instance, the fusion of cortical and blood vessels organoids in Kong et al. (2023) made it possible to demonstrate that, after SARS‐CoV‐2 infection, the assembloids responded with glial activation. It was also notable that these assembloids expressed hyperphosphorylated tau and β‐amyloid plaques, consistent with Alzheimer's disease pathology, reinforcing the hypothesis that neuroinflammation is linked to AD. This system provided an environment containing BBB cells that express angiotensin‐converting enzyme 2 (ACE2) receptor, necessary for viral entry. Additionally, the assembloids combined neuronal and immune cells, which were essential for analyzing the neuroinflammatory response elicited by SARS‐CoV‐2 and for revealing aspects that recapitulate AD pathology (Kong et al. 2023).

Another study, conducted by Stanton et al. (2025), used hiPSCs obtained from AD patients to generate vascular assembloids. Through this model, it was possible to investigate the effects of the classic AD risk variant APOE4. Compared with APOE3‐containing assembloids, APOE4‐containing counterparts exhibited increased levels of reactive astrocyte markers (GFAP, STAT3, C3, and S100β). Notably, these alterations were absent in APOE4 astrocyte monolayers, demonstrating that the reactive phenotype emerged only in the multicellular three‐dimensional environment. Furthermore, the assembloids containing APOE4 astrocytes exhibited elevated hydrogen peroxide and intercellular nitric oxide droplets, lysosomal dysfunction, and increased microglia‐dependent tau phosphorylation (Stanton et al. 2025). Here, it is demonstrated once again the benefits of integrating different cell types in a three‐dimensional culture to unravel processes dependent on the intercellular crosstalk. Moreover, the use of a multidimensional model enabled the investigation of mechanisms triggered by APOE4, a relevant genetic risk factor for AD. In that sense, the assembloid model provides insights into how this variant shapes neuroinflammatory and neurodegenerative pathways.

## 4 Improving Organoid Models to Develop Novel AD Therapies

A potential application of AD organoids is the development of disease‐modifying therapies. Some studies have aimed to validate the model for drug testing by using clinically approved treatments. For instance, tests with the anti‐Aβ monoclonal antibody Lecanemab in vascularized organoids indicates a recapitulation of its clinical effects, showing a significant reduction in amyloid aggregation, associated with increased microglial phagocytosis of Aβ. In addition, activation of endothelial cells was also observed, suggesting impaired vascular integrity, a side effect of Lecanemab (Ji et al. 2025). Likewise, brain organoids can offer insights into the mechanisms of novel compounds, helping to address translational challenges in AD drug development, including therapeutic strategies aiming at inhibiting the production of Aβ by targeting the amyloidogenic pathway. Previous clinical trials have tested β‐secretase inhibitors, but there are open questions regarding their safety and efficacy. In this context, brain organoids may be useful for evaluating the physiological and pathological functions of secretases, as well as the effects of their inhibition on AD‐related alterations. For example, Choe et al. (2024) reported that treating familial AD organoids with β‐ and γ‐secretase inhibitors resulted in a reduction in Aβ and tau burden, as well as decreased apoptosis, indicating the model's suitability for pharmacological testing (Choe et al. 2024).

Importantly, brain organoids also enable drug screening approaches targeting synaptic abnormalities, a central feature of the disease. A study by Ghatak et al. (2021), compared the potential of memantine, used to alleviate neuronal damage in AD, with the synthetic derivative NitroSynapsin, designed to potentiate the inhibition of extrasynaptic NMDA‐type glutamate receptors and thereby reduce excitotoxicity. Electrophysiological analyses in familial AD organoids showed an altered neural network and confirmed that NitroSynapsin reversed neuronal hyperactivity more effectively than memantine. This effect is promising as it appears to reduce synaptic dysfunction in AD (Ghatak et al. 2021). These results indicate that identifying and modulating imbalances in neuronal activity in three‐dimensional human models may open new avenues for therapeutic strategies aimed at delaying cognitive decline.

Different aspects of AD treatment could be investigated in organoids, including neuropsychiatric mechanisms. With this aim, Zivko et al. (2024) developed organoids containing serotonergic neurons, which produced detectable levels of serotonin, to evaluate the response to escitalopram oxalate, a drug usually prescribed to treat depression and agitation in AD. Escitalopram increased serotonin release in a concentration‐dependent manner in both healthy and AD hiPSC‐derived organoids. However, this effect was not observed in all lines tested, indicating inter‐individual variability that may be clinically relevant (Zivko et al. 2024). Similarly, pharmacological approaches targeting AD neuroinflammation have been assessed in three‐dimensional hiPSC models to characterize the response of microglia and other immune cells (Cuní‐López et al. 2024; Gu et al. 2025). For instance, microglia from patients with mild cognitive impairment showed a more robust reduction in cytokine production after treatment with the anti‐inflammatory drug minocycline, compared to microglia from AD patients. The same study pointed out that inter‐patient variability can mask results in cohort analysis, hiding responsive individuals. Furthermore, cytokines indicative of drug effectiveness were identified as potential biomarkers for stratifying patients during screenings (Cuní‐López et al. 2025). Thus, brain organoids can be used as scalable platforms for defining individual therapeutic profiles across different stages of Alzheimer's disease.

Scale‐up attempts have already been made for drug screening using organoids. For example, researchers developed a high‐throughput workflow to test drug repositioning for AD. Using a larger number of organoids, Park et al. (2021) evaluated the efficacy of drugs permeable to the BBB in improving cell viability and reducing the burden of both Aβ and p‐tau within sporadic AD organoids. By employing computational modeling, they were able to identify the most effective drug combinations (Park et al. 2021). However, most pharmacological tests in organoids are limited by small sample sizes. Moreover, future studies should diversify the genetic backgrounds of AD brain organoids to achieve more representative results. Another concern is that hiPSC lines show variability in tau phosphorylation, as well as in the levels of Aβ and APOE secreted by organoids. Moreover, organoids may exhibit differences in terms of cell composition and maturation, cytoarchitecture, and differentiation patterns. These factors hinder batch‐to‐batch reproducibility (Hernández et al. 2022). To overcome these obstacles, standardization and quality control methods have been proposed to improve the accuracy of organoid‐generation protocols (Castiglione et al. 2025).

Another relevant caveat in the use of brain organoids to study AD is their limited degree of cellular maturation. hiPSC‐derived organoids exhibit phenotypes more similar to those of fetal and postnatal stages than to adult brain tissue, as demonstrated by transcriptomic comparisons (Kathuria et al. 2020; Logan et al. 2020). In fact, one of the main applications of brain organoids is to investigate human neurodevelopment (Mulder et al. 2023). Cell maturation in the model progresses gradually and requires the acquisition of a complex cytoarchitecture, which enables the temporal programming of cell fate (Chiaradia et al. 2023). For instance, the electrophysiological properties of brain organoids become more robust over time, indicating that mature neural networks develop only after several months in vitro (Fair et al. 2020). On the other hand, cell viability can be compromised during extended culture periods, which may result in necrotic core formation in larger organoids, making their long‐term maintenance challenging (Mulder et al. 2023; Chen, Sun, et al. 2025). Alternatives for inducing aging in organoids have emerged to better recapitulate features of AD, such as age‐related inflammation (“inflammaging”) and neurodegeneration. Among these, gene perturbation and environmental stress methods for senescence induction stand out (Hossain et al. 2024). Furthermore, the use of cells obtained from aged individuals can be informative. For example, primary monocytes from aged donors have been shown to stimulate the expression of inflammatory and senescence markers in brain organoids (Ao et al. 2022). Thus, incorporating aged peripheral immune cells can contribute to a more representative model of inflammaging.

Given such limitations, using both in vitro and in vivo models in a complementary fashion is considered a valuable strategy to characterize new AD therapeutic candidates. This combination can be advantageous to verify molecular mechanisms in AD organoid and postmortem tissues that correlate with preclinical effects in murine models. Promising new targets have already been identified in this manner through multi‐omics investigations associated with histopathological and behavioral analyses (Xie et al. 2025; Zeng et al. 2025).

Consistent with this view, the transplantation of organoids into mouse brains has emerged as an alternative strategy to overcome key limitations inherent to these systems. As previously described, these constraints include incomplete cellular differentiation and maturation, insufficient tissue perfusion, metabolic restrictions, limited circuit‐level integration, and the lack of immune–vascular interactions (Mansour et al. 2018; Revah et al. 2022). Therefore, this approach enables the investigation of human cells in a complex physiological environment, encompassing increased cellular heterogeneity, vascularization, and integration with sensory and peripheral signals, provided by the host tissue (Paşca et al. 2015). Notably, this strategy promotes the generation of more diverse and mature glial populations, including astrocytic subtypes characteristic of the human brain (Wang et al. 2025). In addition, it also allows the derivation of immunocompetent human microglia, thereby providing an in vivo neuroimmune organoid model suitable to evaluate microglial responses to injury (Schafer et al. 2023). Taken together, xenotransplantation of hiPSC‐derived organoids or assembloids represents a promising approach to expand the experimental toolkit for the development of therapies targeting AD‐associated neuroinflammation.

## 5 Conclusions

The hiPSC‐derived brain organoid model has advanced considerably since its emergence. Strategies for incorporating microglia and vasculature have enhanced its complexity, allowing the study of cell‐to‐cell interactions in a more representative in vitro model of the human brain. This gain in complexity has enabled the emergence of neuroinflammation‐related phenotypes that depend on multicellular organization and that are not captured in two‐dimensional systems. Furthermore, combining patient‐derived brain organoids with genetic tools enables the elucidation of patient‐specific phenotypes, allowing the use of personalized medicine approaches with greater translational relevance than murine systems. These approaches are also advantageous for unraveling neuroinflammation‐associated mechanisms, which can be modulated by both environmental and intrinsic factors, such as APOE isoforms.

Neuroinflammation is considered a hallmark of AD and most likely contributes to disease progression. Microglia play an essential role in this process, as their functional state directly influences protein aggregation, synaptic damage, and neuronal death. Microglia also affect brain homeostasis through their interactions with other resident cells, such as astrocytes, consequently coordinating pathological features of AD (Wilson et al. 2023). As discussed above, several studies have investigated microglial activation and its modulation using hiPSC‐derived brain organoids, considered promising three‐dimensional tools. In the context of AD, modulating microglial activation aiming to promote a more neuroprotective profile might improve disease outcomes. Together, these findings highlight hiPSC‐derived brain organoids as valuable models for improving our understanding of the underlying disease mechanisms and, ultimately, for advancing the development of treatments targeting AD‐related neuroinflammation.

Despite major advances in the field, including the incorporation of microglial and endothelial cells into assembloid systems, as well as improvements in cellular maturation and integration achieved through xenotransplantation, further methodological enhancements are necessary. For instance, improving reproducibility is essential to minimize batch‐to‐batch variation, which can compromise data comparability and weaken the reliability of experimental outcomes. Also, scalability remains a major challenge, as current methods typically generate small numbers of organoids with marked heterogeneity, limiting their applicability in large‐scale studies. Increasing scalability is therefore crucial to boost experimental throughput and ensure the consistent production of uniform organoid structures. Overcoming these issues will substantially improve the robustness of organoid and assembloid platforms for AD drug discovery and high‐throughput screening.

## Author Contributions

**Esther Marques Alves Maciel:** conceptualization, writing – original draft, visualization, validation. **Nathália Costa Silva:** conceptualization, writing – original draft, visualization, validation. **Luiz Gustavo Pontes Santos:** conceptualization, writing – original draft, visualization. **Fabiola M. Ribeiro:** conceptualization, funding acquisition, writing – review and editing, visualization, validation, supervision.

## Funding

This work was supported by Conselho Nacional de Desenvolvimento Científico e Tecnológico (CNPq), grants 403171/2023‐7 and 406968/2024‐1, and Fundação de Amparo à Pesquisa do Estado de Minas Gerais (FAPEMIG) grants APQ‐03921‐22 and APQ‐00140‐23.

## Conflicts of Interest

The authors declare no conflicts of interest.

## Data Availability

The authors have nothing to report.
